# A Multi-Functional Prebiotic Strategy: Crosslinked 2′-Fucosyllactose-Potato Protein Hydrolysate Conjugates Encapsulating Resveratrol for Co-Delivery to Beneficial Gut Bacteria

**DOI:** 10.3390/foods15162923

**Published:** 2026-08-20

**Authors:** Stav Peled, Amit Sontag, Ravit Edelman, Yoav D. Livney

**Affiliations:** 1 Laboratory of Biopolymers for Food and Health, Department of Biotechnology and Food Engineering, Technion-Israel Institute of Technology, Haifa 3200003, Israelravited@technion.ac.il (R.E.); 2 The Esther and Herbert Hecht Sustainable Protein Research Center, Technion-Israel Institute of Technology, Haifa 3200003, Israel

**Keywords:** prebiotic, 2′-fucosyllactose, potato protein, resveratrol, encapsulation, colonic delivery

## Abstract

Prebiotics are predominantly indigestible carbohydrate-based substrates selectively-utilized by beneficial gut-microbes to support host-health. We previously developed protein-containing prebiotics that co-deliver carbohydrate and protein substrates to the colon, where gut-microbes compete for the limited nitrogen availability, thereby enhancing microbial growth, metabolic activity, and host health compared with conventional carbohydrate prebiotics. Resveratrol is a grape-derived polyphenol with antioxidant, anti-inflammatory, and emerging prebiotic activity. Here, we developed a multifunctional protein-containing prebiotic system based on Maillard conjugates of 2′-fucosyllactose–potato protein hydrolysate (2′-FL-PPH) micelles encapsulating resveratrol, followed by genipin crosslinking. This crosslinked 2′-FL-PPH-resveratrol system is designed to limit premature protein and resveratrol absorption, enhancing their colonic co-delivery. We characterized the encapsulation efficacy, physicochemical properties, digestibility and colonic delivery. The conjugates (10 mg/mL 2′-FL-PPH) effectively entrapped resveratrol (600 µM), exhibiting an average particle size of ~28 nm and an encapsulation efficiency of 79.5 ± 4.9%. Binding studies demonstrated predominantly hydrophobic interactions between resveratrol and 2′-FL-PPH. The conjugates prevented resveratrol crystallization in aqueous media, while genipin crosslinking enhanced resistance to simulated gastrointestinal digestion and inhibited premature resveratrol release, increasing the fraction expected to reach the colon. Collectively, this system enables colonic co-delivery of carbohydrate, peptides, and resveratrol, providing a novel strategy for promoting beneficial-microbiota and gut-health.

## 1. Introduction

The human gut microbiome comprises trillions of microorganisms that collectively function as a metabolically active ‘organ’, regulating immune development, pathogen defence, metabolism, essential metabolites biosynthesis, etc. [1]. A balanced and diverse microbiota is widely regarded as essential for maintaining human health, whereas dysbiosis has been linked to metabolic, inflammatory, and other chronic diseases [2].

Among the various factors shaping the gut microbial community, diet plays a pivotal role, and prebiotics are widely recognized as important modulators. Prebiotics are defined as substrates that are selectively utilized by host microorganisms, conferring a health benefit [3]. Their activity depends on selective utilization by specific members of the host microbiota, resulting in changes in microbial composition and/or metabolic activity that are associated with beneficial effects on host physiology [3]. In addition to classical oligosaccharide prebiotics, certain polyphenols are increasingly recognized for their prebiotic properties. Polyphenols are plant-derived secondary metabolites known for their antioxidant, anti-inflammatory, antidiabetic, and anticancer activities. Their prebiotic potential arises from interactions with the gut microbiota, where they modulate composition and metabolism, promote beneficial bacteria, and suppress pathogens [4].

Resveratrol, a stilbenoid polyphenol found in grapes, apples, blueberries, plums, and peanuts [5], is increasingly recognized not only for its antioxidant and anti-inflammatory properties but also for its emerging prebiotic activity [6]. In animal studies, resveratrol supplementation has been shown to enrich beneficial short-chain fatty acids (SCFAs)-producing taxa such as *Blautia*, *Roseburia*, *Lactobacillus*, *Bifidobacterium*, *Akkermansia muciniphila*, Ruminococcaceae, and Lachnospiraceae, while reducing potentially harmful bacteria such as the lipopolysaccharide-producing *Desulfovibrio* [7,8,9]. These microbial shifts are accompanied by increased production of SCFAs, particularly butyrate, which plays a central role in maintaining gut barrier integrity and supporting metabolic health [8,9]. Consistent with these effects, resveratrol has been reported to strengthen intestinal barrier function [9] and plays a key role in controlling obesity through microbiota-mediated mechanisms [10].

Despite these promising biological activities, the effective colonic delivery of resveratrol remains challenging. Clinical studies indicate that the oral absorption of resveratrol is approximately 70–75%, despite its limited aqueous solubility. However, its systemic bioavailability is less than 1% due to its rapid and extensive phase-II metabolism in the intestine and liver [11,12,13,14]. The remaining unabsorbed fraction can reach the colon, where it becomes available for microbial metabolism. Nevertheless, the poor aqueous solubility of resveratrol limits its dispersion and stability in aqueous media [15]. Moreover, given its high degree of absorption in the upper gastrointestinal tract [12], only a limited fraction of orally administered resveratrol is expected to reach the colon in intact form. This provides a rationale for delivery strategies capable of maintaining resveratrol in a soluble state while limiting its premature release and absorption, thereby increasing its potential availability to the beneficial microbes in the colon.

We previously introduced protein-containing prebiotics [16,17,18,19] as a next-generation strategy for beneficial microbiota modulation, designed to supply beneficial commensal colonic bacteria (often used as probiotics) with both oligosaccharide and protein substrates, thereby enhancing their growth compared to the effect of conventional carbohydrate-only prebiotics. These systems are based on Maillard conjugates of prebiotic oligosaccharides and protein hydrolysates, developed to address the microbial competition for colonically-available protein, which is limited due to efficient digestion and absorption of dietary proteins in the small intestine (~90% of intake [20,21]). To improve resistance to small-intestinal digestion and absorption, a dual pre-hydrolysis step using gastric and intestinal proteases is applied prior to Maillard conjugation. The resulting oligosaccharide-peptide conjugates exhibit decreased susceptibility to digestion, thereby increasing their colonic availability, where they are selectively utilized by beneficial microbes. In our recent study [17], we developed 2′-FL-PPH Maillard conjugates that formed micellar structures with good durability to gastrointestinal digestion. This durability substantially increased the fraction of conjugates reaching the colon, where they promoted the growth of beneficial taxa such as *Bifidobacteria* and other SCFAs-producing genera, resulting in elevated colonic SCFAs levels and improved gut and metabolic health compared to conventional prebiotics in mice.

The prebiotic oligosaccharide used in this study, 2′-FL, is the most abundant human milk oligosaccharide and it exhibits well-documented prebiotic, antibacterial, antiviral, and immunomodulatory activities [22]. In addition, 2′-FL has demonstrated benefits for gut and metabolic health [23] and is recognized as GRAS by the FDA [24]. The protein component, potato protein (PP), is derived from an abundant and inexpensive food source, shows very low allergenicity, and is likewise considered GRAS by the FDA [25]. Owing to its water solubility and amphiphilic nature, PP is particularly effective at encapsulating hydrophobic compounds with poor aqueous solubility [26,27]. This property is further complemented by the inherent affinity of polyphenols for proteins, as polyphenols commonly form stable complexes with proteins through hydrophobic interactions and hydrogen bonding [28].

Here, we propose that encapsulating resveratrol within micellar 2′-FL-PPH conjugate complexes and subsequently stabilizing the structures through genipin crosslinking can further limit premature absorption of both the peptide and polyphenol components, thereby increasing the fraction expected to reach the colon intact. Beyond serving as protein-containing prebiotics that deliver carbohydrate and peptide substrates to the colon, the amphiphilic 2′-FL-PPH conjugates also function as carriers for hydrophobic bioactives such as resveratrol. In the colon, resveratrol and the 2′-FL-PPH conjugates are expected to be co-delivered, potentially combining the prebiotic contributions of carbohydrate, peptide, and polyphenol substrates within a single selectively-targeted delivery system. This multi-functional approach may therefore provide enhanced prebiotic activity than each component alone, providing a novel strategy for designing next-generation prebiotic supplements to support gut and metabolic health. Accordingly, the objective of this study was to develop and characterize a genipin-crosslinked 2′-FL-PPH micellar system encapsulating resveratrol and to evaluate its physicochemical properties, gastrointestinal digestibility, and ability to limit premature resveratrol release, with the aim of enhancing the colonic co-delivery of 2′-FL-PPH conjugates and resveratrol.

## 2. Materials and Methods

### 2.1. Materials

2′-FL (94.12%) was purchased from Shandong Deshang Chemical Co., Ltd., Jinan City, China. Potato protein isolate (Solanic 200, 90.5% protein) was purchased from AVEBE, Veendam, The Netherlands. Resveratrol (99%, 430075000, Thermo Scientific Chemicals) was purchased from Rhenium, Modi’in, Israel. All other materials and their source are mentioned in the relevant method section.

### 2.2. Methods

#### 2.2.1. 2′-FL-PPH Preparation

##### Potato Protein Hydrolysis

PPH was prepared as we described previously [17], by sequential digestion of potato protein isolate (Solanic 200, AVEBE; 50 mg/mL) with pepsin (1500 U/mL, pH 3, 1 h, 37 °C) (P7000, Sigma-Aldrich, Rehovot, Israel), followed by trypsin (16,360 U/mL, pH 8, 1 h, 37 °C) (T0303, Sigma-Aldrich, Rehovot, Israel). The hydrolysate was centrifuged (10,000× *g*, 4 °C, 10 min), and the supernatant was freeze-dried.

##### 2′-FL and PPH Conjugation

2′-FL-PPH conjugates were prepared using the Maillard reaction as we described previously [17]. Briefly, PPH and 2′-FL solution (20 mg/mL PPH and 100 mg/mL 2′-FL in distilled water (DW)) was adjusted to pH 8 and incubated at 80 °C for 3 h. The solution was filtered (0.22 µm), dialyzed (1 kDa cut-off, 24 h), and freeze-dried to obtain 2′-FL-PPH powder.

#### 2.2.2. Encapsulation of Resveratrol in 2′-FL-PPH Conjugates

The encapsulation was performed according to a protocol described previously by our group [27], with slight modifications. 2′-FL-PPH conjugates (10 mg/mL in DW) were stirred while ethanolic resveratrol solution was added dropwise. Control samples included 2′-FL-PPH alone and resveratrol alone, prepared under identical conditions. All samples contained 2.2% ethanol and were stirred for equilibration at room temperature for 1 h.

#### 2.2.3. Particle Size Distribution Analysis by Dynamic Light Scattering (DLS)

Particle size distribution of 2′-FL-PPH-resveratrol complexes (10–1300 µM resveratrol), 2′-FL-PPH alone (10 mg/mL), and resveratrol alone (600 µM) were analyzed by DLS following sample preparation as described above (Section 2.2.2). Measurements were performed using a Zetasizer Ultra (Malvern Instruments Ltd., Malvern, UK), at a size range of 1–10,000 nm.

#### 2.2.4. Zeta (ζ) Potential Analysis

ζ-potential of 2′-FL-PPH, 2′-FL-PPH-resveratrol complexes, and resveratrol solutions were measured using Zetasizer Ultra (Malvern Instruments Ltd., Malvern, UK), to assess colloidal stability. Samples (10 mg/mL 2′-FL-PPH and 600 µM resveratrol) were prepared as described above (Section 2.2.2) and diluted 5-fold with DW before measurement.

#### 2.2.5. Encapsulation Efficiency and Loading Capacity

Encapsulation efficiency and loading capacity were quantified as described previously, with slight modifications [29]. The encapsulated resveratrol was quantified indirectly by measuring unbound resveratrol. 2′-FL-PPH (10 mg/mL) samples containing increasing resveratrol concentrations (10 µM–1300 µM) were prepared as described above (Section 2.2.2), and centrifuged (12,000× *g*) at 4 °C for 20 min. The pellet containing unencapsulated resveratrol was lyophilized, dissolved in ethanol, vortexed for 15 min, and centrifuged (10,000× *g*, 10 min, 4 °C). Resveratrol in the supernatant was analyzed by Reversed-Phase High-performance liquid chromatography (HPLC) as described below (Section 2.2.15). Resveratrol concentration was determined using a calibration curve (0.8–14 µg/mL). Encapsulated resveratrol was calculated by subtracting unencapsulated resveratrol from the total amount added. Encapsulation efficiency and loading capacity were calculated as demonstrated in Equations (1) and (2):

(1) $$Encapsulation\;efficiency\;\left(\%\right)=\frac{m_{res,enc}\;}{m_{res,add}}\cdot 100\%$$

(2) $$Loading\;capacity\;\left(\frac{\mu g}{mg\;conjugates}\right)=\frac{m_{res,enc}}{m_{con}}$$

m_res,add_ is the mass of resveratrol added to the sample, m_res,enc_ is the mass of encapsulated resveratrol, and m_con_ is the mass of the conjugates in the sample.

#### 2.2.6. Binding Studies by Ultraviolet-Visible (UV-Vis) Absorbance Spectra

UV-Vis absorbance spectroscopy was used to qualitatively examine the interaction between resveratrol and 2′-FL-PPH, as described previously [30]. 2′-FL-PPH-resveratrol complexes (10 mg/mL 2′-FL-PPH, 600 µM resveratrol) were prepared as described above (Section 2.2.2) and diluted 40-fold with DW before measurement. Control samples of 2′-FL-PPH and resveratrol were prepared at equivalent final concentrations (0.25 mg/mL and 15 µM, respectively). Binding was qualitatively evaluated by comparing the absorbance spectrum of the complex with the sum of the individual spectra. Measurements were performed using a UV-Vis spectrophotometer (Evolution 201, Thermo Scientific, Madison, WI, USA).

#### 2.2.7. Fourier Transform Infrared Spectroscopy (FTIR) Analysis

FTIR analysis was performed as described previously [31], and was used as a qualitative spectroscopic approach to investigate the non-covalent interactions involved in resveratrol association with 2′-FL-PPH. The 2′-FL-PPH-resveratrol complexes (10 mg/mL 2′-FL-PPH and 600 µM resveratrol) were prepared as described above (Section 2.2.2), stirred for 1 h and centrifuged (12,000× *g*, 20 min, 4 °C) to remove unencapsulated resveratrol. The supernatant was lyophilized. Mid IR (400–4000 cm^−1^ wavenumber) transmittance spectra of 2′-FL-PPH, 2′-FL-PPH-resveratrol complexes, and resveratrol alone were recorded on a Bruker Tensor 27 FTIR spectrometer (Bruker Optics GmbH & Co. KG, Ettlingen, Germany) equipped with an attenuated total reflectance (ATR) diamond crystal. The spectra were recorded with 72 scans and a 4 cm^−1^ resolution. All spectra were baseline-corrected.

#### 2.2.8. Light Microscopy Imaging

Light microscopy was used for qualitative assessment of resveratrol crystallization in the presence and absence of 2′-FL-PPH (10 mg/mL) over a concentration range of 0–1300 µM. Samples were prepared as described above (Section 2.2.2) and imaged after 1 h at room temperature. Images were captured at 60×magnification using an Olympus BX51 light microscope (Olympus Optical Co., Ltd., Tokyo, Japan) in bright-field or polarized light optical mode.

#### 2.2.9. Genipin Crosslinking

Genipin crosslinking was performed as described previously [32,33], with slight modifications. 2′-FL-PPH-resveratrol complexes were prepared as described above (Section 2.2.2). Genipin (466642500, Thermo Scientific, purchased from Rhenium, Modi’in, Israel), dissolved in ethanol, was then added to obtain final concentrations of 0–12 mM, a range selected based on concentrations previously reported in the literature for protein crosslinking with genipin [34]. Samples were stirred in the dark at room temperature for 24 h. Aliquots were collected at 0 and 24 h, snap-frozen in liquid nitrogen, and stored at −20 °C.

#### 2.2.10. Crosslinking Analysis

##### UV-VIS Spectrometry

Blue color, developed due to the crosslinking reaction between genipin and amine groups [35,36,37], was studied to monitor the extent of crosslinking. Crosslinked 2′-FL-PPH-resveratrol complexes (at 0 and 24 h) were diluted 20-fold, loaded into a 96-well plate, and absorbance at 600 nm was measured using a UV-visible spectrophotometer.

##### Free Amine Groups

The extent of crosslinking was quantified by measuring changes in free amine groups [32] using the 2,4,6-trinitrobenzenesulfonic acid (TNBS) method as described previously [38]. Samples were diluted with 0.1 M sodium bicarbonate buffer (pH 8.5) to 100 µg/mL protein. 0.5 mL of each sample was reacted with 0.25 mL of 0.01% (*w*/*v*) TNBS (P2297, Sigma-Aldrich, Rehovot, Israel) solution at 37 °C for 2 h. Then, 0.25 mL of 10% SDS and 0.125 mL of 1 N HCl were added to each sample. Absorbance at 335 nm was measured using a UV-visible spectrophotometer. Free amine groups (%) were calculated as described in Equation (3):

(3) $$Free\;amine\;groups\;\left(\%\right)=\;\frac{A_{f}}{A_{0}}\times 100$$

where $A_{f}$ is the absorbance following 24 h of crosslinking reaction with 2–12 mM genipin, and $A_{0}$ is the absorbance following 24 h stirring with 0 mM genipin.

#### 2.2.11. Sodium Dodecyl Sulfate-Polyacrylamide Gel Electrophoresis (SDS-PAGE)

Genipin crosslinking of 2′-FL-PPH-resveratrol complexes and changes in the molecular-weight distribution of their digestion products during in vitro simulated gastrointestinal digestion were qualitatively assessed using SDS-PAGE, as described previously [18], with slight modifications. Samples were normalized for protein content, mixed with reducing loading buffer, heated (100 °C, 5 min), and loaded (20 µL) onto 15% acrylamide gels. Electrophoresis was performed at 120 V (stacking gel) followed by 180 V (resolving gel). Gels were fixed, stained with Coomassie Brilliant Blue R-250, and destained.

#### 2.2.12. Cryogenic Transmission Electron Microscopy (Cryo-TEM)

Cryo-TEM imaging of 2′-FL-PPH (10 mg/mL), 2′-FL-PPH-resveratrol complexes (10 mg/mL 2′-FL-PPH and 600 µM resveratrol) and crosslinked 2′-FL-PPH-resveratrol complexes (10 mg/mL 2′-FL-PPH, 600 µM resveratrol, and 2 or 4 mM genipin), prepared as described above (Section 2.2.2 and Section 2.2.9), was performed. Cryo-TEM imaging was performed by applying a 3 µL droplet onto a perforated carbon film supported on a standard TEM grid (Ted Pella, Redding, CA, USA). This process was performed in the GP2 (Leica, Wetzlar, Germany) under ambient temperature, saturated with water vapor [39]. The specimen was vitrified by rapid plunging into liquid ethane at −183 °C and subsequently loaded into a TEM cooling holder (“cryo-stage”) using a dedicated “transfer station”. Throughout imaging, the holder tip was maintained at approximately −180 °C to prevent sublimation and ensure preservation of the supercooled state of the vitreous ice. The imaging was performed with the minimal electron dose possible (about 10 e^−^/Å^2^) on Talos F200X FEG TEM (Thermo Fisher) using a “Volta phase plate” for contrast enhancement [40]. The micrographs were recorded with a direct Falcon electron camera through TEM Imaging & Analysis (TIA) software, version 5.0 SP4. In this study, cryo-TEM was used for qualitative morphological characterization of the micellar structures rather than for quantitative analysis of morphological micelle populations.

#### 2.2.13. In Vitro Simulated Gastrointestinal Digestion

In vitro simulated digestion of 2′-FL-PPH-resveratrol, genipin-crosslinked 2′-FL-PPH-resveratrol, and resveratrol in DW was performed to assess digestibility and resveratrol release, using a protocol described previously [41]. Samples were prepared as described above (Section 2.2.2 and Section 2.2.9; 10 mg/mL 2′-FL-PPH, 40 µM resveratrol; genipin 2 mM or 4 mM), freeze-dried, and reconstituted to 100 mg/mL conjugates and 400 µM resveratrol. At this concentration, resveratrol remained fully soluble throughout the gastric and intestinal phases when prepared in DW (≤200 µM), enabling a comparable resveratrol-only control. Samples were subjected to gastric digestion (simulated gastric fluid, pepsin 2000 U/mL, CaCl_2_ 0.075 mM, pH 3, 37 °C, 2 h) and then intestinal digestion (simulated intestinal fluid, trypsin 100 U/mL, chymotrypsin (C4129, Sigma-Aldrich, Rehovot, Israel) 25 U/mL, bile salts (5 mM taurocholic acid sodium salt hydrate (T4009, Sigma-Aldrich, Rehovot, Israel), 5 mM sodium glycodeoxycholate ((G9910, Sigma-Aldrich, Rehovot, Israel), CaCl_2_ 0.3 mM, pH 7, 37 °C, 2 h). Aliquots were taken during digestion; the enzymatic activity was stopped using Pefabloc^®^ SC (PEFBSC-RO, Roche; purchased from Sigma-Aldrich, Rehovot, Israel) (1 mM); samples were snap-frozen in liquid nitrogen and kept at −20 °C for further analysis.

#### 2.2.14. Resveratrol Release upon Digestion

Following in vitro simulated gastrointestinal digestion, resveratrol release was evaluated as described previously for polyphenols bioaccessibility [42], with slight modifications. Digested samples (0.5 mL) were filtered through a 3 kDa Amicon^®^ ultra centrifugal filter tube (Merck Millipore, Darmstadt, Germany) (16,100× *g*, 25 °C, for 25 min). The filtrate was collected, and filters were inverted and centrifuged (1000× *g*, 2 min) to remove the retentate, containing the colonically-available fraction. As the Amicon^®^ membranes can adsorb polyphenols, leading to underestimated release [42], a washing step was incorporated. Filters were washed with 0.4 mL ethanol:DW (1:1) and centrifuged (16,100× *g*, 25 °C, 25 min). Filtrates from both steps were combined, their final volume was measured (±10 µL), filtered (0.22 µm), and resveratrol content (i.e., released resveratrol) was quantified by HPLC.

#### 2.2.15. HPLC Analysis

Resveratrol was quantified as described previously [43], with slight modifications. Detection was performed at 307 nm using an HP Agilent 1100 HPLC system (Agilent Technologies, Waldbronn, Germany) equipped with a diode array detector (DAD) and a Bio-Basic™ 18 column (Thermo Scientific). Mobile phases A (25% ACN, 60% HPLC-grade water, 15% MeOH) and B (37.5% ACN, 32.5% HPLC-grade water, and 30% MeOH) were applied with a programmed gradient (A: 0–3.5 min; B: 3.6–5.8 min; A: 5.9–15 min) at a flow rate of 1 mL/min and an injection volume of 20 µL.

#### 2.2.16. Statistical Analysis

All experiments were performed at least in duplicates, with most performed in triplicates or more. Data is shown as means ± SE of the mean. Differences among samples were analyzed using one-way analysis of variance (ANOVA) followed by Tukey’s HSD post hoc test. Differences were considered statistically significant at *p*-values ≤ 0.05. The Origin 2019b software (OriginLab Corporation, Northampton, MA, USA) and MS Excel were used for data processing and visualization.

## 3. Results and Discussion

### 3.1. Particle Size Distribution and ζ-Potential

**Figure 1:**
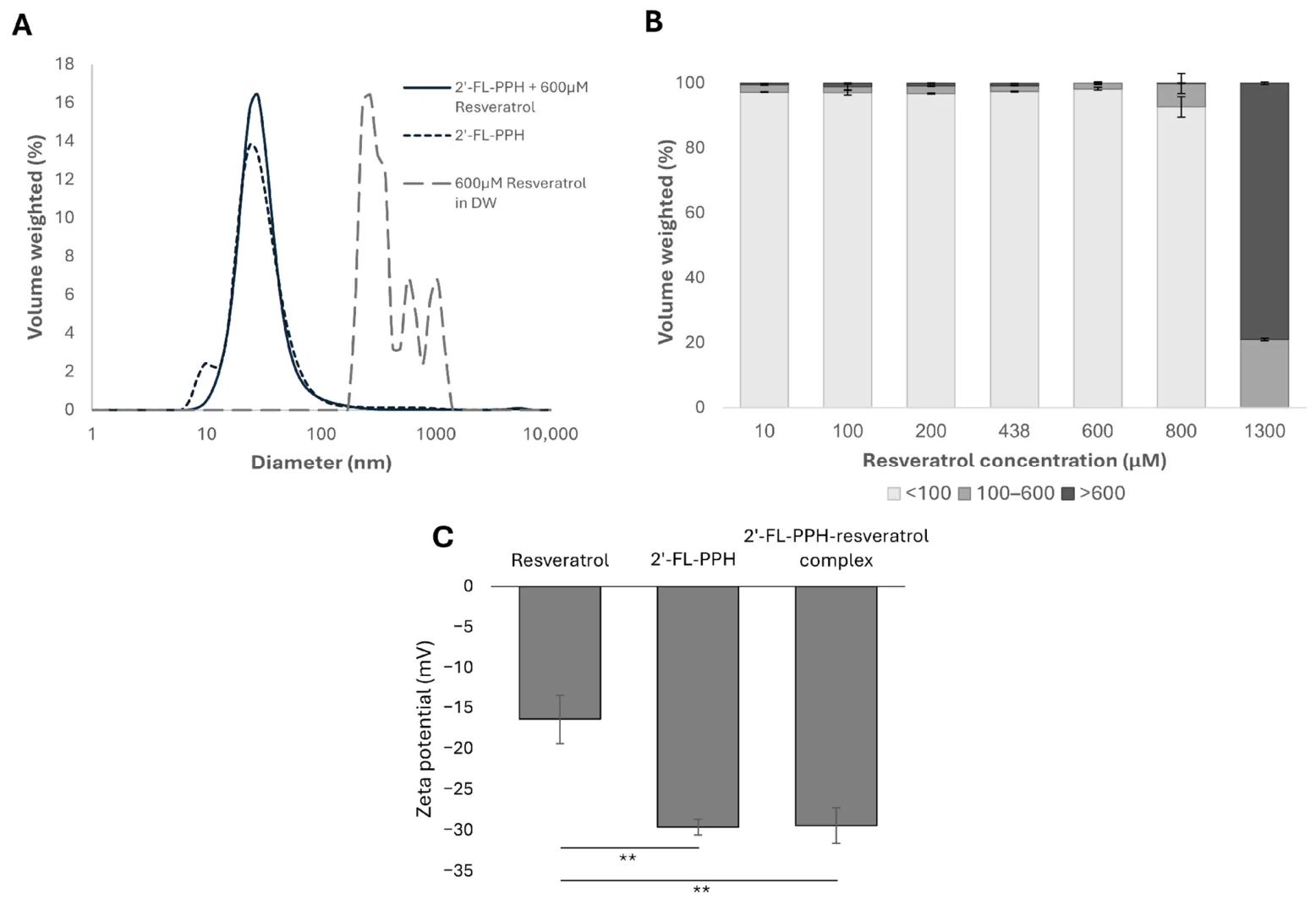
Particle size distribution: (**A**) Volume-weighted size distribution of 2′-FL-PPH-resveratrol complex (10 mg/mL 2′-FL-PPH, 600 µM resveratrol), 2′-FL-PPH (10 mg/mL), and resveratrol in DW (600 µM). Data are presented as the means of 4 independent samples, each measured in triplicate. (**B**) Percent of volume-weighted size by size range (<100 nm, 100–600 nm, >600 nm) as a function of resveratrol concentration (10 µM–1300 µM), at a constant concentration of 2′-FL-PPH (10 mg/mL). Data are presented as mean ± SE of 2 independent samples, each measured in triplicate. (**C**) Zeta potential (mV) measurements of resveratrol, 2′-FL-PPH and 2′-FL-PPH-resveratrol complex. Samples were prepared in DW at 10 mg/mL of 2′-FL-PPH, and 600 µM resveratrol, followed by 5-fold dilution in DW. Data are presented as mean ± SE of 4 independent samples, each measured in triplicate. ** *p* < 0.01.

Size distribution analysis of the 2′-FL-PPH-resveratrol complexes and those of the 2′-FL-PPH and resveratrol alone, was performed using DLS, as shown in Figure 1A.

The size distribution observed for 2′-FL-PPH included a small population of ~10 nm particles, and a larger population of ~24 nm. For resveratrol alone in DW (600 µM), three populations with diameters of ~267 nm, ~568 nm, and ~1039 nm were observed, indicating its low solubility in DW. When resveratrol was added to the 2′-FL-PPH solution, the average particle diameter decreased (compared to resveratrol in DW), showing only 1 particle population of ~28 nm, demonstrating the effective entrapment of resveratrol by 2′-FL-PPH conjugates. Size distribution of 2′-FL-PPH-resveratrol complexes was evaluated across increasing resveratrol concentrations (10–1300 µM), while maintaining a constant 2′-FL-PPH concentration of 10 mg/mL (Figure 1B). Particles smaller than 100 nm (predominantly 20–30 nm) were observed at resveratrol concentrations up to 600 µM. At 800 µM, most particles (92.7% ± 3.1%) remained below 100 nm; however, a minor population (7.2% ± 3.0%) of larger particles (100–600 nm) was detected, presumably indicating the onset of the encapsulation capacity limit. At the highest resveratrol concentration (1300 µM), the amount of resveratrol exceeded the encapsulation capacity of the 2′-FL-PPH conjugates, leaving unbound resveratrol that promoted the formation of large aggregates. Hence, it can be deduced from these results that 2′-FL-PPH at a concentration of 10 mg/mL effectively encapsulates resveratrol up to a resveratrol concentration of 600 µM. ζ-Potential values are shown in Figure 1C. Unencapsulated resveratrol exhibited a ζ-potential of −16.4 ± 3 mV, reflecting a relatively unstable system. In contrast, 2′-FL-PPH-resveratrol complex and 2′-FL-PPH alone displayed ζ-potentials of −29.4 ± 2.2 mV and −29.6 ± 1.0 mV, respectively, indicative of colloidally stable particles, consistent with their small size. The charge imparted by 2′-FL-PPH to resveratrol upon entrapment promotes electrostatic repulsion, resulting in improved dispersion into smaller, more stable particles.

### 3.2. Encapsulation Efficiency and Loading Capacity

**Figure 2:**
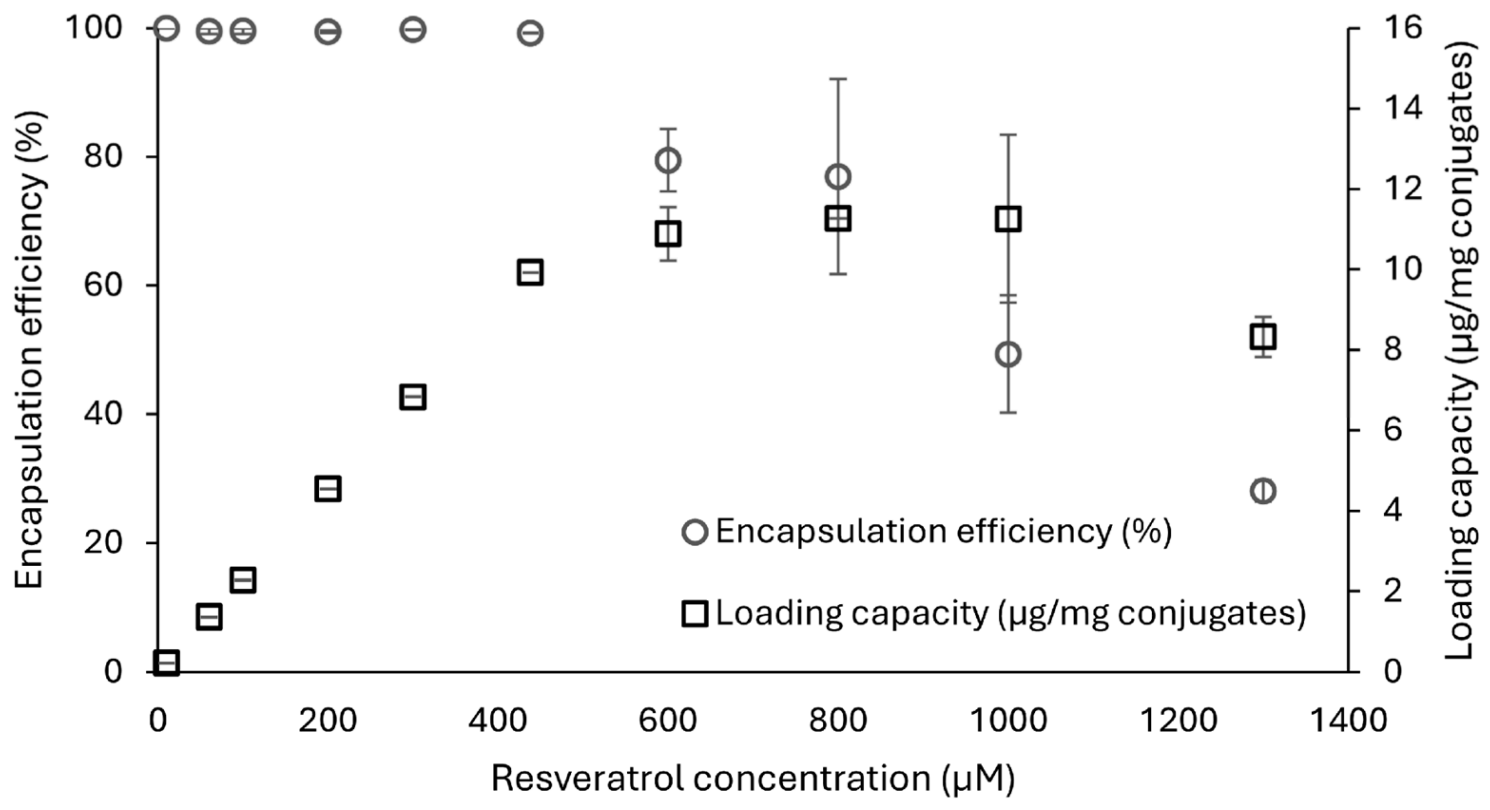
Encapsulation efficiency and loading capacity of resveratrol in 2′-FL-PPH conjugates at increasing resveratrol:2′-FL-PPH ratio. 2′-FL-PPH concentration: 10 mg/mL, resveratrol concentration: 10 µM–1300 µM. Values presented are means ± SE of 4 independent experiments.

The loading capacity and encapsulation efficiency of resveratrol in 2′-FL-PPH conjugates were assessed at increasing resveratrol concentrations (10–1300 µM), while maintaining a constant 2′-FL-PPH concentration (10 mg/mL). Unbound resveratrol was separated by centrifugal sedimentation and quantified using HPLC (Figure 2).

As expected, increasing the resveratrol-to-2′-FL-PPH ratio resulted in a reduction in the encapsulation efficiency, while the loading capacity increased until reaching a maximum. As shown in Figure 2, the loading capacity increased with increasing resveratrol concentration, with no substantial increase beyond 600 µM. In addition, beyond 800 µM, encapsulation efficiency dropped sharply, coinciding with the appearance of a minor fraction of larger particles (100–600 nm, 7.2% ± 3.0%) in the DLS analysis (Figure 1B), indicating the onset of the encapsulation capacity limit. Considering loading capacity, encapsulation efficiency, and particle size together, the optimal formulation was identified as 600 µM resveratrol with 10 mg/mL 2′-FL-PPH, providing the highest payload while maintaining good encapsulation efficiency. Under these conditions, the complexes exhibited a loading capacity of 10.9 ± 0.7 µg resveratrol/mg conjugates and an encapsulation efficiency of 79.5 ± 4.9%. The ability of the 2′-FL-PPH conjugates to achieve both high encapsulation efficiency and loading capacity highlights their potential as carriers for poorly water-soluble bioactives.

### 3.3. Binding Studies

**Figure 3:**
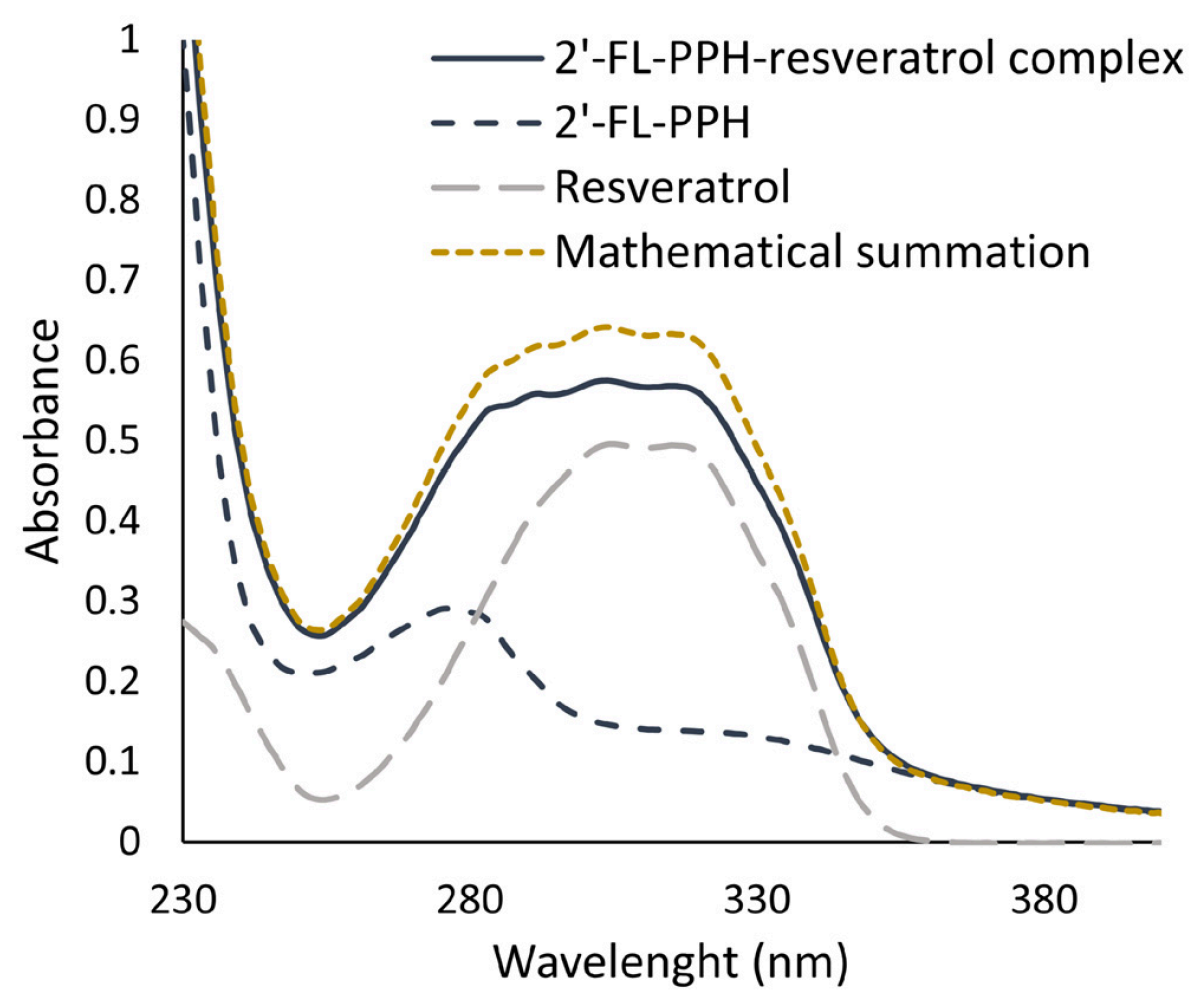
Absorbance spectra (230 nm–400 nm) of non-encapsulated resveratrol (15 µM in DW), 2′-FL-PPH conjugates at 0.25 mg/mL, and of resveratrol (15 µM) encapsulated in 2′-FL-PPH conjugates at 0.25 mg/mL, compared to a mathematical summation of the absorbance spectra of non-encapsulated resveratrol and of the conjugate solution. Absorbance spectra are presented as the mean of 4 independent experiments.

UV absorbance spectra were used to qualitatively assess the interaction between resveratrol and 2′-FL-PPH. Figure 3 presents the spectra of 15 µM resveratrol and 0.25 mg/mL 2′-FL-PPH separately, alongside that of their mixture at the same final concentrations. The mathematical sum of the individual spectra was calculated and plotted for comparison.

In the absence of interaction, the spectrum of the mixture is expected to match the calculated sum of the spectra. However, as shown in Figure 3, the absorbance of resveratrol and 2′-FL-PPH mixture was lower than the sum of the two individual spectra. This deviation from spectral additivity provides qualitative evidence for an interaction between resveratrol and the 2′-FL-PPH conjugates, similarly to previous observations from our lab [26,30].

**Figure 4:**
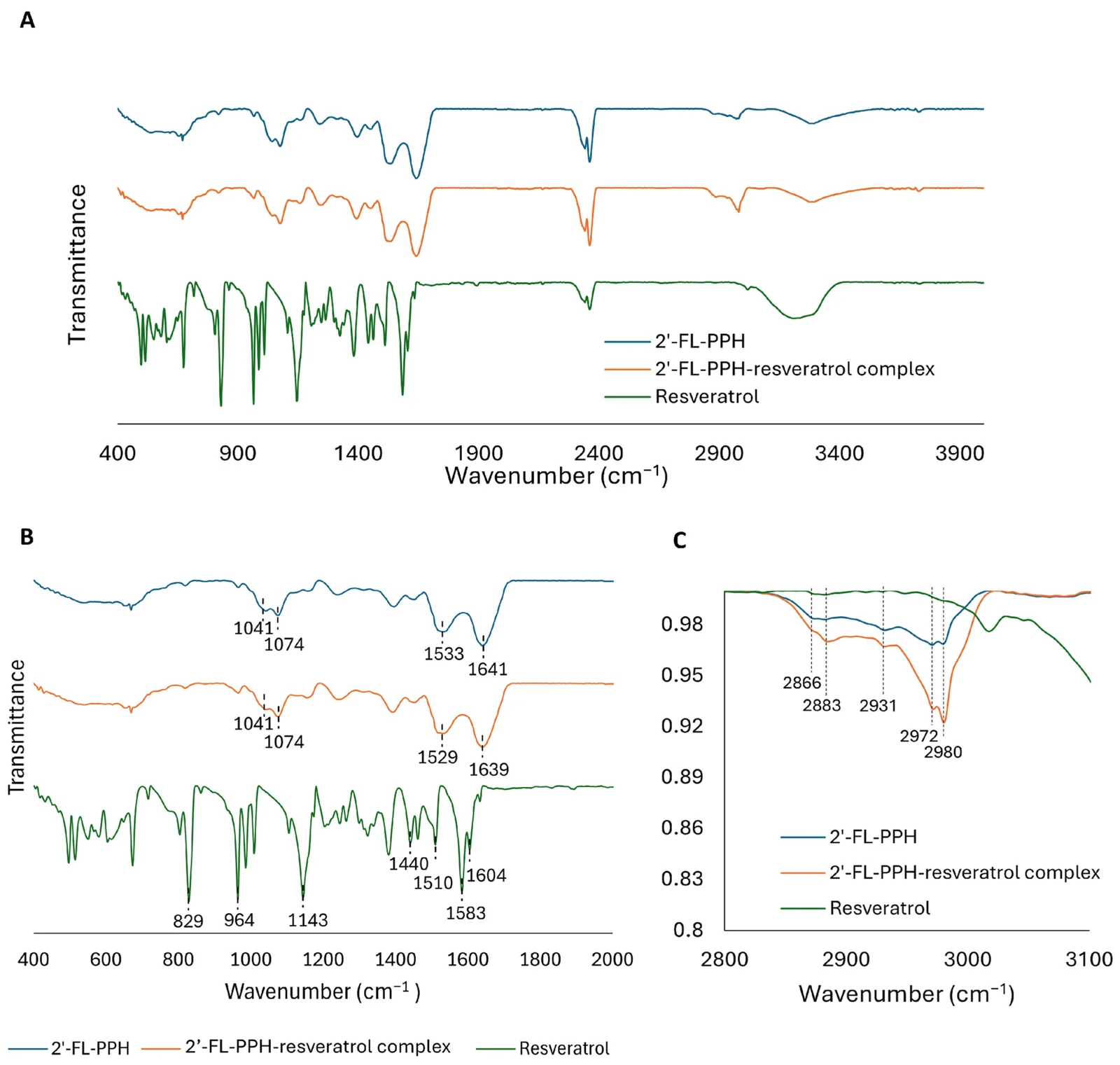
(**A**) ATR-FTIR transmittance spectra of dried 2′-FL-PPH, 2′-FL-PPH complexes with resveratrol, and resveratrol alone. (**B**) Magnified spectral region from 400–2000 cm^−1^ wavenumber and (**C**) magnified spectral region from 2800–3100 cm^−1^, with vertical markers indicating characteristic absorption bands. Data are presented as the means of 4 independent samples.

FTIR analysis was performed to examine the interactions between 2′-FL-PPH and resveratrol. Therefore, FTIR spectra of 2′-FL-PPH, 2′-FL-PPH-resveratrol complex and resveratrol alone were measured (Figure 4A).

The characteristic transmittance peaks of the 2′-FL-PPH conjugates were observed at 1641 cm^−1^, 1533 cm^−1^, and 1074&1041 cm^−1^ wavenumbers (Figure 4B), corresponding to amide I (C=O stretching vibration of the amide group [44]), amide II [45] (C–N stretching coupled to N–H bending vibration of the amide group [44]), and C–O–C stretching vibration [44], respectively. Free resveratrol exhibited distinctive peaks at 1604 cm^−1^, 1583 cm^−1^, 1510 cm^−1^, 1440 cm^−1^, 1143 cm^−1^, 964 cm^−1^, and 829 cm^−1^ (Figure 4B). The adjacent peaks at 1604 and 1583 cm^−1^ are attributed to C=C aromatic double-bond stretching and C–C olefinic stretching [46,47], while the peaks at 1510 cm^−1^ and 1440 cm^−1^ correspond to benzene skeleton vibrations [31]. The peak at 964 cm^−1^ is assigned to the bending vibration of C=C–H [46].

Minor shifts in the amide I and amide II peaks were observed upon resveratrol encapsulation (Figure 4B), suggesting slight changes in PPH secondary structure [48]. The most prominent spectral changes appeared in the 2800–3000 cm^−1^ region, where the 2′-FL-PPH-resveratrol complex showed reduced transmittance at 2866, 2883, 2931, 2972, and 2980 cm^−1^ relative to 2′-FL-PPH alone (Figure 4C). These peaks correspond to CH_2_ and CH_3_ stretching vibrations [49], supporting the involvement of hydrophobic interactions between resveratrol to hydrophobic regions of PPH. This aligns with previous reports attributing spectral changes in this region to hydrophobic binding between resveratrol and proteins [49]. Such protein-polyphenol interactions are primarily mediated by non-covalent hydrophobic associations and further stabilized by hydrogen bonding [28].

### 3.4. Light Microscopy

**Figure 5:**
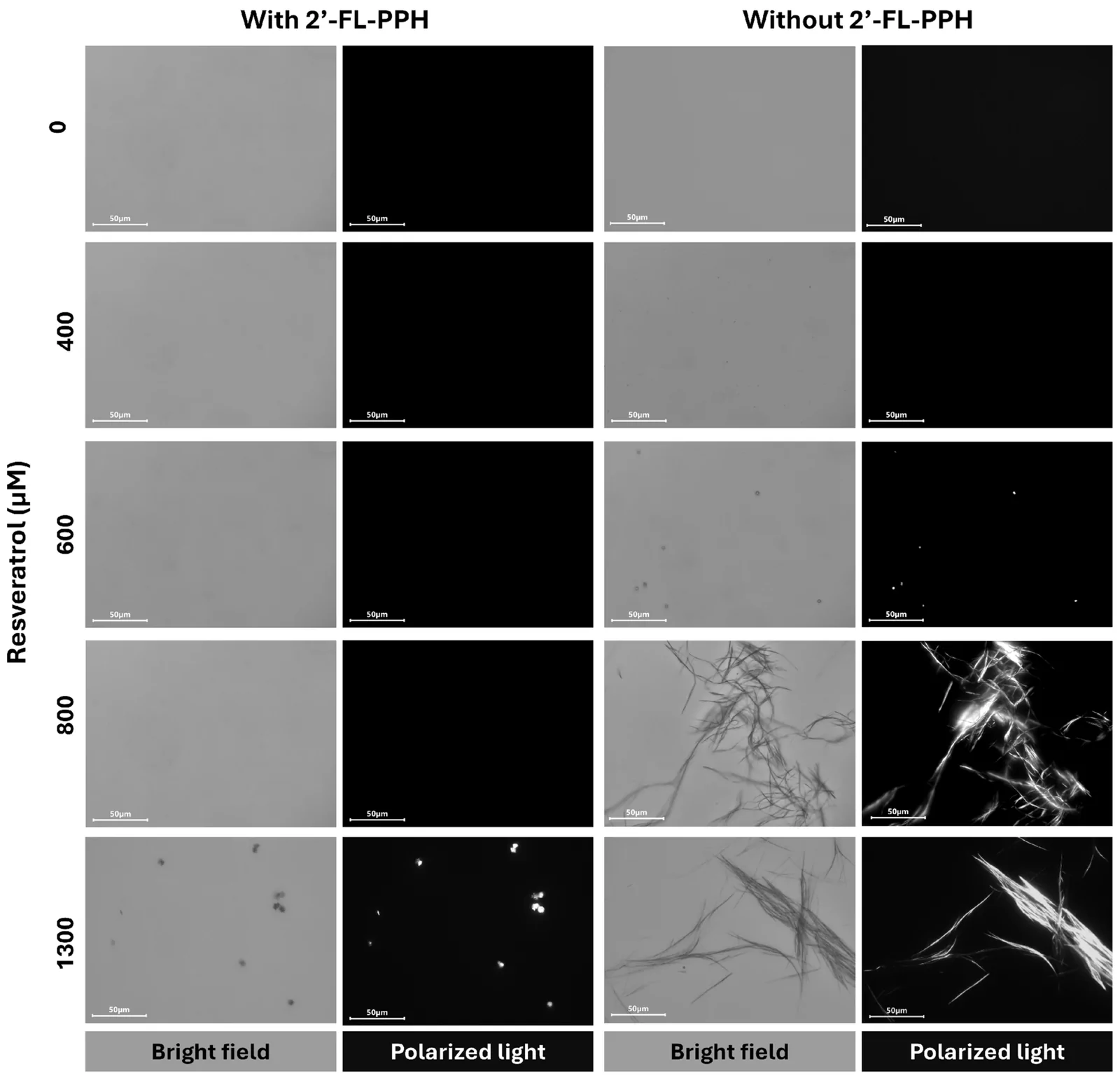
Light microscopy images of resveratrol (0, 400, 600, 800, and 1300 μM) with or without 2′-FL-PPH (10 mg/mL), captured under bright-field and polarized-light modes. Scale bar: 50 μm.

To further investigate the encapsulation of resveratrol by 2′-FL-PPH in aqueous solution, light microscopy was performed as a qualitative assessment of resveratrol aggregation and crystallization in samples containing resveratrol (0–1300 μM) with and without 2′-FL-PPH (10 mg/mL) (Figure 5).

Resveratrol alone formed visible aggregates from 400 µM, with aggregates appearing progressively larger with concentration and exhibiting light polarization at ≥600 µM, indicating crystallinity. This is in agreement with a previous study reporting the high inherent crystallization tendency of resveratrol, attributed to its three hydroxyl groups forming an extensive hydrogen-bonding network and its planar structure, which together result in a high melting point (262 °C) and a small number of low-energy conformers, thus promoting crystallization [50]. In contrast, no visible crystals were observed in the presence of 2′-FL-PPH up to 800 µM, consistent with particle size data showing that most particles remained below 100 nm (Figure 1B). At 1300 µM, visible small crystals appeared, suggesting this concentration exceeds the encapsulation capacity. Notably, crystals observed in the presence of 2′-FL-PPH appeared qualitatively smaller and morphologically different from those formed by resveratrol alone, supporting both the attractive interaction between the conjugates and resveratrol, and its effective encapsulation up to 600–800 µM resveratrol. It is proposed that 2′-FL-PPH, particularly its protein fraction (PPH), provides hydrogen-bonding sites that compete with resveratrol-resveratrol interactions, while hydrophobic interactions further promote resveratrol-2′-FL-PPH association over resveratrol self-interaction, thereby limiting crystal growth.

### 3.5. Genipin Crosslinking

**Figure 6:**
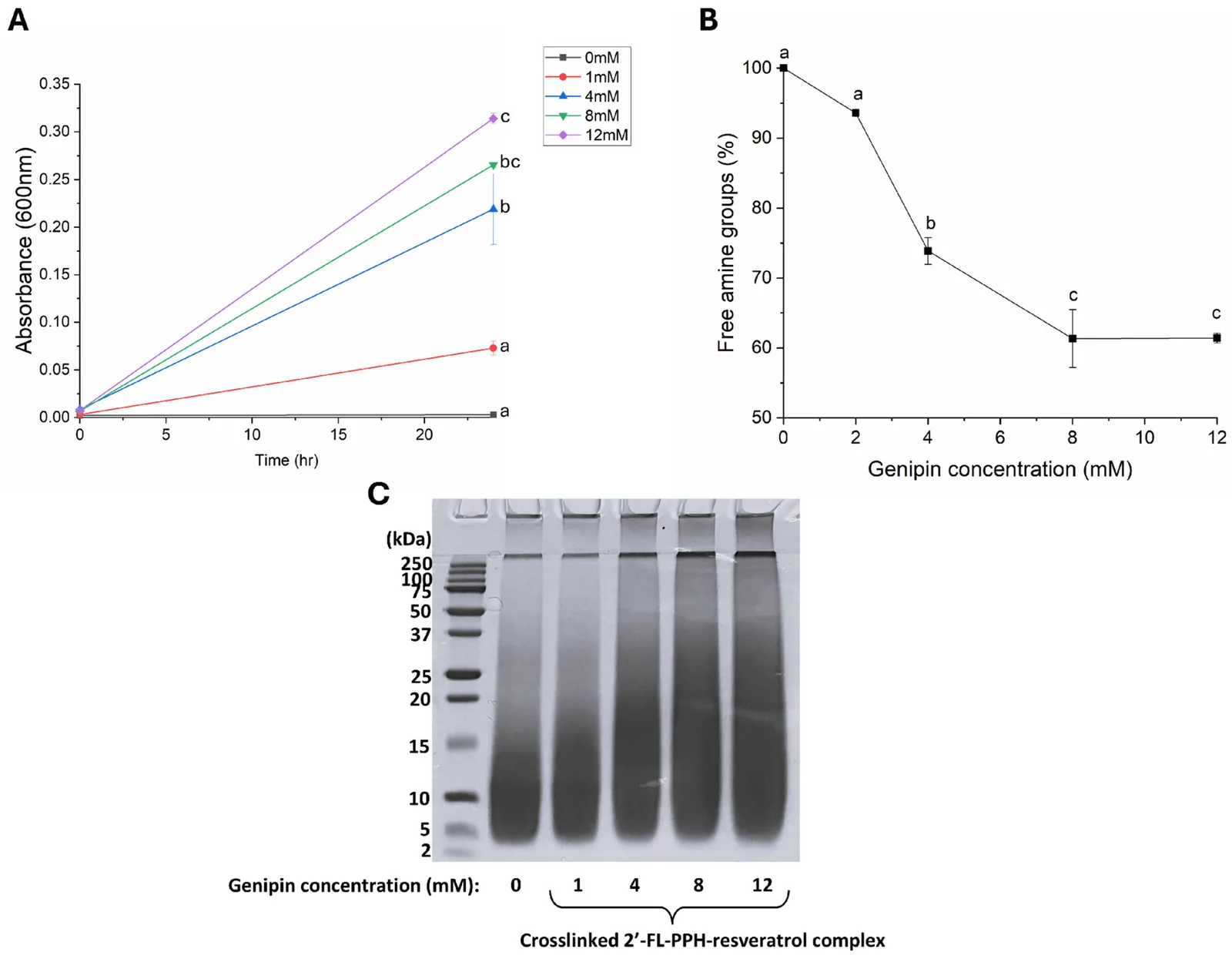
Genipin crosslinking analysis. (**A**) Absorbance at 600 nm of 2′-FL-PPH-resveratrol complexes crosslinked with increasing genipin concentrations (0–12 mM). Samples were diluted 20-fold prior to measurement. (**B**) Percentage of free amine groups in 2′-FL-PPH-resveratrol complexes after crosslinking, determined by the TNBS method. Different letters indicate statistically significant differences between groups (*p* < 0.05). (**C**) SDS-PAGE analysis of 2′-FL-PPH-resveratrol complexes crosslinked with genipin (0–12 mM). Complexes contained 10 mg/mL 2′-FL-PPH and 40 µM resveratrol. Crosslinking was carried out at room temperature for 24 h.

To facilitate colonic delivery of the intact 2′-FL-PPH-resveratrol complex and to develop a multifunctional prebiotic system with enhanced activity relative to each component individually, the complex was crosslinked with genipin. Genipin is a bioactive aglycone extracted from the gardenia fruit [51], traditionally used in herbal medicine and as a natural food colorant [52]. Previous studies have reported its low toxicity [52] and anti-inflammatory properties [44]. As a crosslinker, genipin readily reacts with protein amine groups, forming a blue chromophore [53] and generating a semi-interpenetrating protein network [44]. This crosslinked structure may protect the protein from proteolytic degradation [53], improve colonic delivery, and limit premature release of encapsulated resveratrol. Evaluation of crosslinking is presented in Figure 6.

As shown in Figure 6A, absorbance at 600 nm increased with rising genipin concentration after 24 h, reflecting formation of the characteristic blue chromophore, indicating a higher degree of crosslinking. These findings are consistent with previous studies linking increased blue color intensity to greater crosslinking degree in protein solutions [53,54]. To further quantify crosslinking, free amine groups were measured using the TNBS assay. Genipin treatment caused a dose-dependent reduction in free amine up to 8 mM, reaching a 38.7% ± 4.1% decrease (Figure 6B), in agreement with earlier reports observing a reduction in free amine groups upon genipin-crosslinking [32,55,56]. SDS-PAGE analysis was conducted to further assess the extent of crosslinking, as an increase in molecular weight is expected with higher degrees of crosslinking. SDS-PAGE analysis supported these findings, showing a shift toward higher-molecular-weight species at ≥4 mM genipin (Figure 6C), consistent with progressive protein crosslinking [32]. As the 12 mM genipin-crosslinked conjugates showed negligible digestion by SDS-PAGE, lower genipin concentrations (2 and 4 mM) were selected for further analysis to allow partial digestion and to better distinguish concentration-dependent effects on digestibility.

Beyond its structural role in controlling digestibility and resveratrol release, genipin may also contribute direct bioactive benefits within the gut environment. Recent work demonstrated that genipin alleviates colitis in mice by suppressing inflammatory and oxidative responses [57]. Together with the established gut-health effects of 2′-FL-PPH [17] and the anti-inflammatory and microbiota-modulating properties of resveratrol [6], this crosslinked system may offer a dual advantage: enhanced prebiotic delivery alongside amplified therapeutic potential for mitigating gut microbiota-related inflammatory diseases, such as inflammatory bowel diseases.

### 3.6. Cryo-TEM

**Figure 7:**
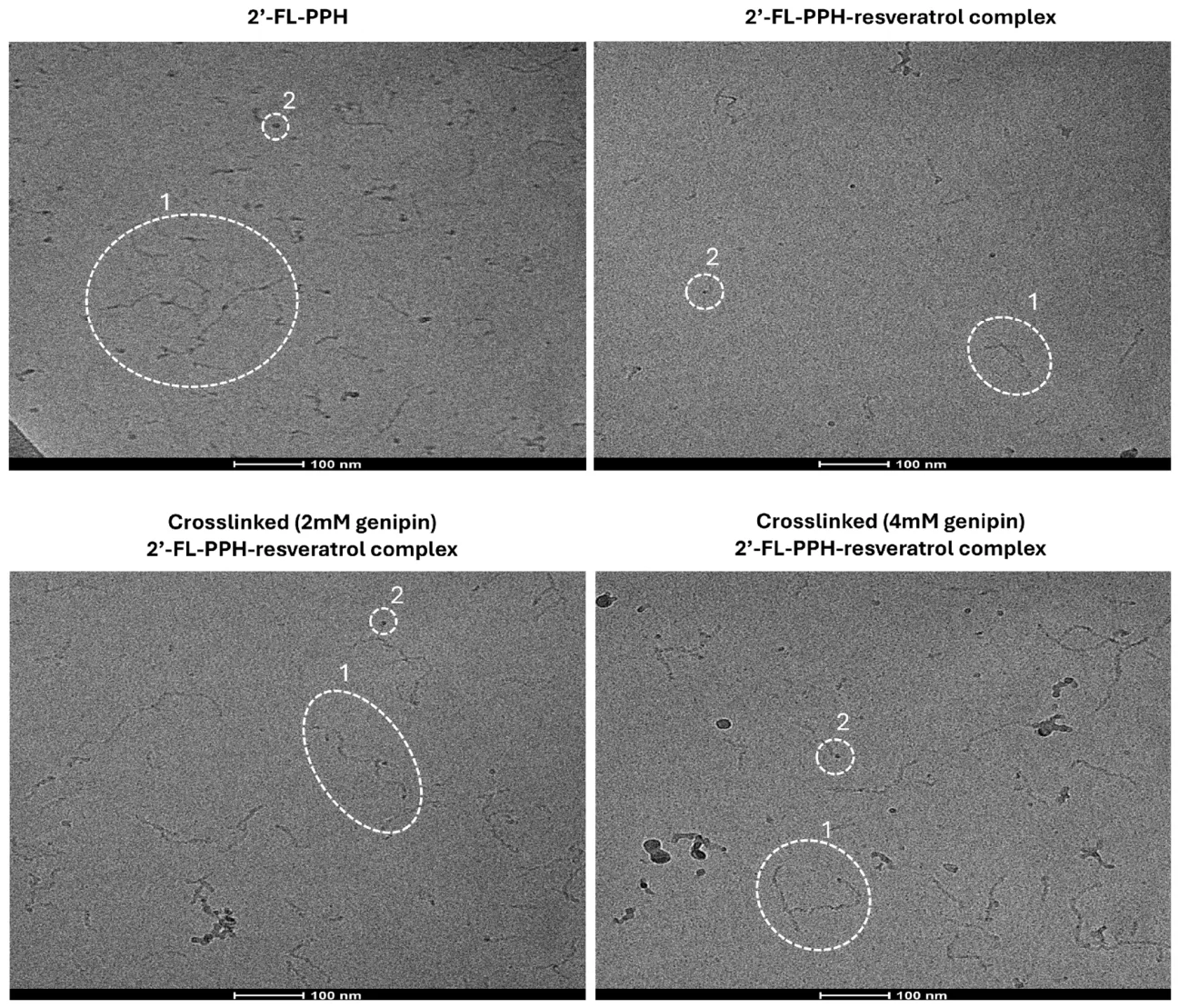
Cryo-TEM images of 2′-FL-PPH, 2′-FL-PPH-resveratrol complex, and crosslinked complexes treated with 2 and 4 mM genipin. 2′-FL-PPH concentration was 10 mg/mL and resveratrol concentration was 600 µM. Two typical particle types are highlighted with dashed white circles: (1) worm-like micelles and (2) individual spherical micelles.

The structures formed by 2′-FL-PPH, 2′-FL-PPH-resveratrol complexes, and complexes crosslinked with 2 and 4 mM genipin were examined using Cryo-TEM (Figure 7).

2′-FL-PPH self-assembles into micellar structures, owing to its amphiphilic nature arising from the amphiphilicity of PPH in addition to the hydrophilic conjugated oligosaccharides. As we previously reported [17], 2′-FL-PPH self-assembles into two types of micellar particles: worm-like micelles (1) and small spherical micelles (2). The equilibrium between these structures is dependent on the packing parameter, with a higher packing parameter value favoring the formation of worm-like micelles [58]. A higher packing parameter is expected to result from larger peptides conjugated to 2′-FL. Representative cryo-TEM images showed that both spherical and worm-like micellar structures were present before and after resveratrol encapsulation and genipin crosslinking, with no obvious qualitative changes in overall micellar morphology. This observation suggests that low-level genipin crosslinking preserves the overall micellar architecture while potentially reinforcing micellar integrity and improving resistance to digestive enzyme penetration.

### 3.7. Digestibility

**Figure 8:**
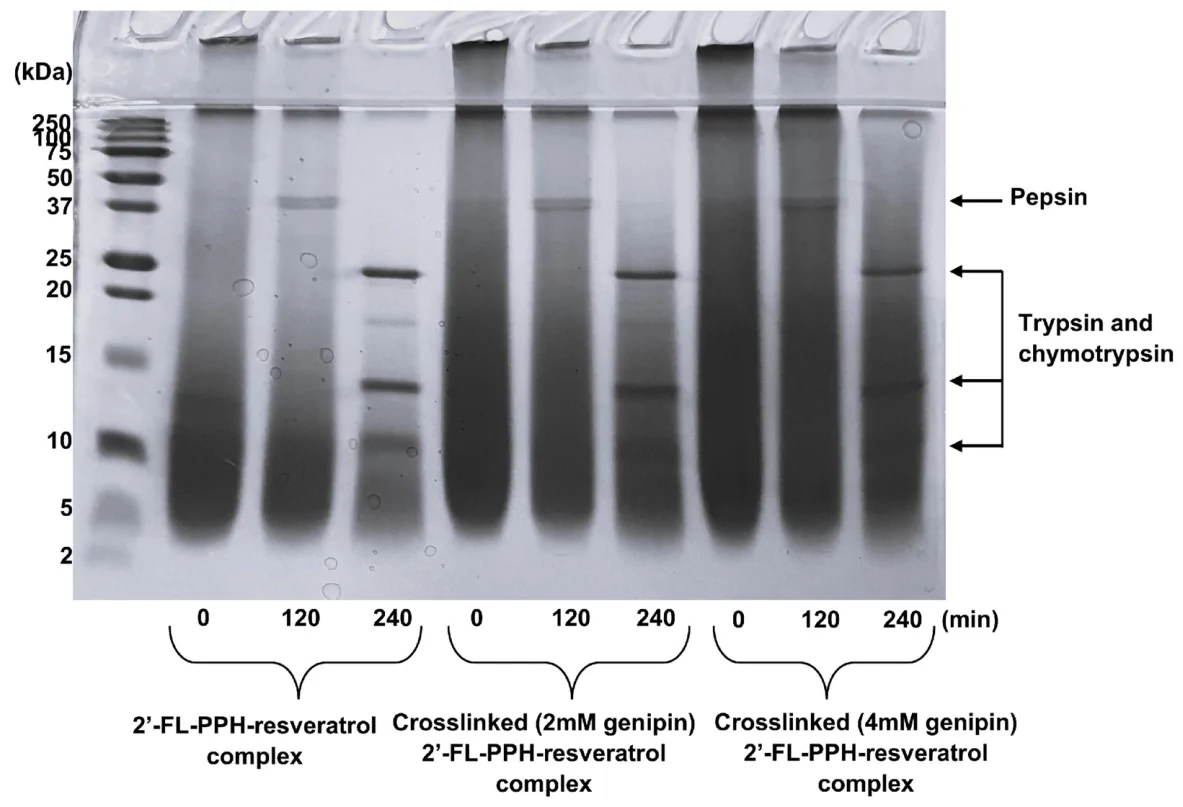
SDS-PAGE of in vitro simulated gastrointestinal digestion of 2′-FL-PPH, 2′-FL-PPH-resveratrol complexes and crosslinked 2′-FL-PPH-resveratrol complexes (with 2 mM and 4 mM genipin). Numbers below each lane indicate digestion time (min): 120 min corresponds to 2 h of gastric phase; 240 min corresponds to the subsequent 2 h of intestinal phase.

The digestibility of 2′-FL-PPH, 2′-FL-PPH-resveratrol complexes, and complexes crosslinked with 2 and 4 mM genipin was evaluated using in vitro simulated gastrointestinal digestion and qualitatively analyzed by SDS-PAGE (Figure 8), to assess changes in proteolysis-resistance and peptide molecular-weight distribution, as indicators of the potential for colonic-delivery. Based on crosslinking characterization and preliminary digestion experiments, the 2 and 4 mM genipin were selected for subsequent digestion analyses as these concentrations provided intermediate degrees of crosslinking while still allowing observable proteolytic degradation during simulated gastrointestinal digestion.

The non-crosslinked complex exhibited relatively low digestibility, with residual peptides mainly in the 5–25 kDa range following full digestion. Previous studies reported that peptides with a molecular weight larger than 3.5–5 kDa are not absorbed via the paracellular route or the di-/tripeptide active transporters [59,60,61], displaying a low intestinal permeability rate [61,62]. Accordingly, these peptides are expected to have a high probability of reaching the colon, remaining available to gut microbes. The SDS-PAGE profiles indicated reduced proteolytic degradation following genipin-crosslinking, with greater persistence of high-molecular-weight species up to ~250 kDa at the higher genipin concentration, consistent with the known proteolysis-resistance of genipin-crosslinked proteins [51,63]. Mechanistically, pepsin and chymotrypsin preferentially cleave peptide bonds adjacent to hydrophobic/aromatic residues, whereas trypsin targets the carboxyl side of lysine and arginine [64,65,66]. It is suggested that the dense and compact protein network formed by genipin crosslinking impedes pepsin and chymotrypsin penetration into the hydrophobic micellar protein core, reducing exposure of aromatic and hydrophobic cleavage sites, thereby impairing enzyme–substrate binding and subsequent proteolysis. In parallel, modification of amine residues of basic amino acids, such as lysine and arginine, during crosslinking [67], reduces the accessibility of trypsin to its cleavage sites. The resulting interpeptide covalent crosslinks are also expected to be resistant to digestion by gastrointestinal proteases [63]. Thereby, genipin crosslinking enhances proteolysis-resistance and particle stability under the gastrointestinal digestion environment, thus making it a promising platform for designing orally administered colonic delivery systems.

### 3.8. Resveratrol Release upon Digestion

**Figure 9:**
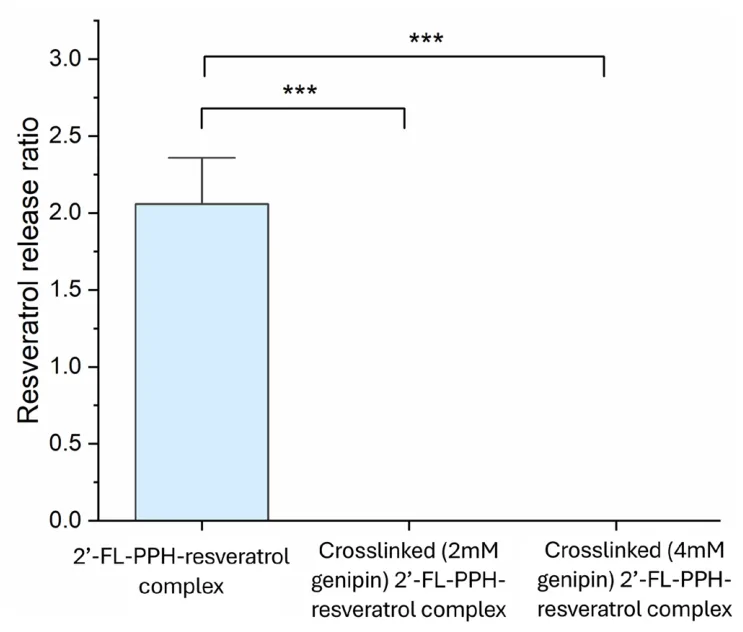
Resveratrol release ratio, compared to the release of resveratrol prepared in DW, following full simulated gastrointestinal digestion of 2′-FL-PPH-resveratrol complex and crosslinked 2′-FL-PPH-resveratrol complexes (with 2 mM and 4 mM genipin). *** *p* < 0.001.

Figure 9 shows the in vitro release of resveratrol from the 2′-FL-PPH-resveratrol complexes and their counterparts crosslinked by 2 mM and 4 mM genipin, after simulated gastrointestinal digestion. Release values were calculated relative to the release measured for resveratrol prepared in DW under identical digestion conditions. Resveratrol release was assessed using Amicon^®^ ultra centrifugal filter tubes (3 kDa molecular weight cut-off) (Merck Millipore, Darmstadt, Germany). The filtrate is expected to contain free resveratrol and resveratrol bound to small peptides (<3 kDa), which are small enough to cross the intestinal epithelium and be absorbed. The retentate represents a potentially colonically-available pool, containing resveratrol bound to larger peptides (>3 kDa) with a low likelihood of small-intestinal absorption. Because both free resveratrol and resveratrol associated with peptides <3 kDa were considered potentially absorbable in the small intestine, these species were analyzed together as the ‘released fraction’. Resveratrol concentration in the intestinal phase was 100 µM, a concentration at which resveratrol is fully dissolved in the digestion matrix, even without encapsulating materials, thus precluding low release of resveratrol due to its self-aggregation.

As shown in Figure 9, resveratrol encapsulated within 2′-FL-PPH exhibited a ~2-fold higher release after complete digestion, compared to its release when prepared in DW. A previous study reported in vitro low bioaccessibility of polyphenols during the intestinal phase due to bile binding [42], which is suggested to be governed by hydrogen bonds [68]. As described by Eran Nagar et al. (2023) [42], bile binding markedly reduces polyphenol bioaccessibility, especially in purified matrices with few competing bile-binding constituents, such as resveratrol in DW. It is suggested that the enhanced release from the 2′-FL-PPH complexes may therefore reflect competitive binding of resveratrol between bile acids and 2′-FL-PPH conjugates, reducing bile-resveratrol association, thus subsequently facilitating release of resveratrol from the matrix following digestion.

Due to the enhanced release of resveratrol from the 2′-FL-PPH-resveratrol complexes, compared to the purified matrix (resveratrol dissolved in DW), crosslinking was used to retain resveratrol within the complex, attenuate premature resveratrol release, and thereby increase the colonically-available fraction. In addition, genipin-crosslinking markedly reduced 2′-FL-PPH digestibility, increasing the proportion of conjugates expectedly reaching the colon and, collectively, is expected to enhance prebiotic activity relative to either component alone. A previous study reported that digestible biopolymer carriers tend to release resveratrol in the stomach or small intestine, whereas indigestible carriers primarily release it in the colon, via microbiota-mediated degradation [69]. As shown in Figure 9, crosslinking with 2 mM and 4 mM genipin significantly inhibited resveratrol release from the complex upon digestion, attributed to the major increase in conjugate durability under simulated gastric and intestinal digestion environments. These observations are consistent with previous reports, showing reduced polyphenol release from protein particles upon genipin crosslinking [44,53]. It should be noted that previous studies reported that genipin does not inhibit the growth of beneficial commensal bacteria such as bifidobacteria in the range of 0–25 mM [70], making it a suitable crosslinker in the current prebiotic delivery system.

Based on these promising findings, several aspects warrant further investigation. The present study did not directly evaluate the effects of the crosslinked system on gut microbial growth, community composition, or metabolic activity. In addition, the proposed enhancement of colonic delivery was inferred from in vitro gastrointestinal digestibility and resveratrol-release experiments, and should be validated in vivo. It also remains important to determine whether the encapsulated resveratrol is accessible to beneficial gut bacteria upon arrival to the colon. Future studies should therefore include microbiota-based fermentation and/or in vivo investigations to establish the colonic fate, microbial utilization, and biological functionality of the developed system.

## 4. Conclusions

In this study, we developed and characterized a multifunctional prebiotic delivery system based on 2′-FL-PPH conjugates forming micellar complexes encapsulating resveratrol and stabilized by genipin crosslinking. The conjugates effectively entrapped resveratrol, forming stable micellar structures. Genipin-crosslinking enhanced the structural integrity of the complexes without altering micellar morphology, markedly reduced susceptibility to gastrointestinal proteolysis, and significantly attenuated premature resveratrol release during simulated digestion. Consequently, a larger fraction of both the protein-containing prebiotic conjugates and resveratrol is expected to reach the colon. Importantly, both components contribute health-promoting functions: 2′-FL-PPH beneficially modulates the gut microbiota, enhancing intestinal barrier function, and improving gut and metabolic health in vivo, while resveratrol is known for its anti-inflammatory and beneficial microbiota-supporting effects. Moreover, genipin itself possesses anti-inflammatory activity, suggesting an added therapeutic dimension. Together, this multi-functional system provides synchronized targeted co-delivery of carbohydrate, peptide, and polyphenol substrates to beneficial commensal colonic bacteria, thereby expected to exhibit boosted prebiotic activity, while simultaneously offering potential anti-inflammatory benefits. Overall, this work presents a next-generation prebiotic platform that integrates enhanced colonic delivery with multiple bioactivities, offering a promising strategy for promoting gut and metabolic health and for mitigating microbiota-related inflammatory conditions. Thus, the findings presented in this work provide interesting insights into the development of functional foods and novel prebiotic formulations. Future research will evaluate the prebiotic activity and health benefits of this system in vivo, compared to conventional and protein-containing prebiotics.

## Data Availability

The raw data supporting the conclusions of this article will be made available by the authors on request.

## Abbreviations

The following abbreviations are used in this manuscript:

2′-FL-PPH	2′-fucosyllactose–potato protein hydrolysate (conjugates)
SCFA	Short-chain fatty acid
PP	potato protein
2′-FL	2′-fucosyllactose
PPH	Potato protein hydrolysate
DW	distilled water
DLS	Dynamic light scattering
HPLC	High-performance liquid chromatography
UV-vis	Ultraviolet-visible (light spectroscopy)
FTIR	Fourier transform infrared spectroscopy
ATR	Attenuated total reflectance
TNBS	2,4,6-trinitrobenzenesulfonic acid
SDS-PAGE	Sodium dodecyl sulfate-polyacrylamide gel electrophoresis
Cryo-TEM	Cryogenic transmission electron microscopy
DAD	Diode array detector
ANOVA	(One-way) analysis of variance
